# Geospatial Transmission Hotspots of Recent HIV Infection — Malawi, October 2019–March 2020

**DOI:** 10.15585/mmwr.mm7109a1

**Published:** 2022-03-04

**Authors:** Carson T. Telford, Zaena Tessema, Malango Msukwa, Melissa M. Arons, Joe Theu, Fred Fredrick Bangara, Alexandra Ernst, Susie Welty, Gabrielle O’Malley, Trudy Dobbs, Vedapuri Shanmugam, Alinune Kabaghe, Helen Dale, Nellie Wadonda-Kabondo, Salem Gugsa, Andrea Kim, George Bello, Jeffrey W. Eaton, Andreas Jahn, Rose Nyirenda, Bharat S. Parekh, Ray W. Shiraishi, Evelyn Kim, James L. Tobias, Kathryn G. Curran, Danielle Payne, Andrew F. Auld

**Affiliations:** ^1^Division of Global HIV & TB, Center for Global Health, CDC; ^2^Public Health Institute, Oakland, California; ^3^International Training and Education Center for Health, Lilongwe, Malawi; ^4^Global Strategic Information, Institute for Global Health Sciences, University of California, San Francisco, California; ^5^International Training and Education Center for Health, Department of Global Health, University of Washington, Seattle, Washington; ^6^Division of Global HIV & TB, Center for Global Health, CDC, Lilongwe, Malawi; ^7^Department of HIV & AIDS, Ministry of Health, Lilongwe, Malawi; ^8^Medical Research Council Centre for Global Infectious Disease Analysis, School of Public Health, Imperial College London, London, United Kingdom.

Persons infected with HIV are more likely to transmit the virus during the early stages (acute and recent) of infection, when viral load is elevated and opportunities to implement risk reduction are limited because persons are typically unaware of their status (*1*,*2*). Identifying recent HIV infections (acquired within the preceding 12 months)* is critical to understanding the factors and geographic areas associated with transmission to strengthen program intervention, including treatment and prevention (*2*). During June 2019, a novel recent infection surveillance initiative was integrated into routine HIV testing services in Malawi, a landlocked country in southeastern Africa with one of the world’s highest prevalences of HIV infection.^†^ The objectives of this initiative were to collect data on new HIV diagnoses, characterize the epidemic, and guide public health response (*2*). New HIV diagnoses were classified as recent infections based on a testing algorithm that included results from the rapid test for recent infection (RTRI)^§^ and HIV viral load testing (*3*,*4*). Among 9,168 persons aged ≥15 years with a new HIV diagnosis who received testing across 103 facilities during October 2019–March 2020, a total of 304 (3.3%) were classified as having a recent infection. Higher proportions of recent infections were detected among females, persons aged <30 years, and clients at maternal and child health and youth clinics. Using a software application that analyzes clustering in spatially referenced data, transmission hotspots were identified with rates of recent infection that were significantly higher than expected. These near real-time HIV surveillance data highlighted locations across Malawi, allowing HIV program stakeholders to assess program gaps and improve access to HIV testing, prevention, and treatment services. Hotspot investigation information could be used to tailor HIV testing, prevention, and treatment to ultimately interrupt transmission. 

During June 2019, a phased approach was used to integrate recent infection surveillance into HIV testing services in 11 of Malawi’s 28 districts. For persons aged ≥13 years who received a new HIV diagnosis and consented to recent infection surveillance, providers performed a finger prick to conduct an RTRI using the Asante HIV-1 Rapid Recency Assay (Sedia Biosciences). If RTRI results indicated recent infection, additional specimens were collected for viral load testing. Using the testing algorithm for recent infections, new diagnoses were classified as recent if RTRI results indicated a recent infection and viral load was ≥1,000 copies/mL. 

This analysis included 103 facilities in five districts that carried out surveillance activities during October 2019-March 2020. These districts were selected based on availability of HIV-testing data disaggregated by age and sex at the facility level. Among 9,295 persons with a new diagnosis of HIV during this period meeting eligibility criteria, 127 (1.4%) declined participation. Two persons aged <15 years were excluded to prevent inclusion of persons who might have been infected through mother-to-child transmission; another four persons who self-reported a previous HIV diagnosis or antiretroviral therapy (ART), were also excluded. In addition, 252 persons whose RTRI results indicated recent infection with a viral load <1,000 copies/mL were excluded because viral suppression likely indicated previous ART use and HIV diagnosis. 

Transmission hotspots were defined as one or more facilities in which the observed rate of recent infections was significantly higher (p<0.05) than the expected rate. Transmission hotspot analysis was conducted using SaTScan software (version 9.6; Harvard Medical School) via spatial scan statistic in a Poisson probability model to identify clustering of facilities, using facility geographic coordinates, with significantly higher diagnosis rates of recent HIV infection compared with what was expected based on the overall rate of recent infection (*5*,*6*). Rates of recent infection were calculated as the number of recent HIV infections per 100,000 persons at risk for HIV (total number of recent infections plus the total number of negative HIV tests). Relative risks were calculated as the risk for recent infection among facilities inside a given hotspot compared with the risk outside of the hotspot. Facilities reported the total number of HIV negative test results quarterly. Because not all facilities were collecting recent infection surveillance data by October 1, 2019, the number of total HIV-negative tests was adjusted proportionally, assuming testing uniformity across quarters. Hotspots were ranked by probability of occurrence based on log-likelihood and reported using the letter “P.” The analysis was adjusted for sex and age (<30 years or ≥30 years), and did not allow cluster overlap.^¶^ A maximum cluster radius (20 kilometers [12.4 miles]) was selected to identify smaller hotspots and to allow response efforts to focus on facilities that contributed most to high rates of recent infection. Given its population density, a secondary analysis was conducted in Blantyre district in the southern region of the country, with a maximum radius of 5 kilometers (3.1 miles), to identify potential micro-hotspots within this district. Hotspots from this secondary analysis were also ranked according to the log-likelihood and reported using the letter “S.” Statistical analyses were conducted using R (version 3.5.0; R Foundation) to analyze the percentage of recent HIV infections among total tests performed, by district, age group, sex, HIV testing service entry point, and facility urban-rural classification. This activity was reviewed and approved by the Malawi National Health Science Research Institutional Review Board and was reviewed by CDC and conducted consistent with applicable federal law and CDC policy.**

Among 9,168 new HIV diagnoses, 3.3% (304) were recent infections (Table 1). The number of recent infections was highest in Blantyre district (116). The percentages of new diagnoses that were recent infections was highest in Machinga district (6.9%) and lowest in Blantyre (2.4%) and Mangochi (2.4%) districts. The percentage of new diagnoses that were recent infections was highest among persons aged <30 years (4.6%), females (4.0%), clients at youth clinics (12.8%) and maternal/child health clinics (6.3%) and those who received a diagnosis in rural facilities (3.8%).

**TABLE 1:** Demographic characteristics of persons with new HIV diagnoses at health facilities implementing recent HIV infection surveillance―Malawi, October 2019−March 2020

Characteristic	No. of new HIV diagnoses	No. of recent infections (%)
**Overall**	9,168	304 (3.3)
**District**		
Blantyre	4,770	116 (2.4)
Lilongwe	945	48 (5.1)
Machinga	1,057	73 (6.9)
Mangochi	801	19 (2.4)
Zomba	1,595	48 (3.0)
**Age group, yrs**		
<30	3,871	177 (4.6)
≥30	5,297	127 (2.4)
**Sex**		
Male	3,655	85 (2.3)
Female	5,513	219 (4.0)
**Entry point**		
HTC or VCT	5,724	179 (3.1)
Antenatal care	820	27 (3.3)
Inpatient department	560	15 (2.7)
Maternal and child health	80	5 (6.3)
Outpatient department	1,604	51 (3.2)
Youth clinic	86	11 (12.8)
Other	294	16 (5.4)
**Residence**		
Urban	4,979	147 (3.0)
Rural	4,165	157 (3.8)
Unknown	24	0 (—)

**Abbreviations:** HTC = HIV testing and counseling; VCT = voluntary counseling and testing.

Spatial analyses identified six transmission hotspots: three in the primary analysis (P) and three in the secondary analysis (S) (Table 2) (Figure). In the primary analysis, the median age (range = 26–30 years) of persons with recent infection was similar across hotspots. Hotspot P1 was in Blantyre district, a mostly urban area^††^ that includes four facilities within a radius of 10.2 kilometers (6.3 miles). The recent infection rate in Blantyre district was 575 per 100,000 persons at risk for HIV (relative risk [RR] = 3.1; p<0.001); the highest percentage of recent infections occurred among females (81.5%). Hotspot P2 included four facilities located across the border of Machinga and Zomba districts within a radius of 16.1 kilometers (9.9 miles), but were primarily in Machinga district, a mostly rural area. The recent infection rate in hotspot P2 was 376 per 100,000 persons at risk (RR = 2.0; p = 0.018). Hotspot P3 was a single facility in Blantyre with a recent infection rate of 818 per 100,000 persons at risk (RR = 4.2; p = 0.025). In the secondary analysis limited to Blantyre district, hotspot S1 included two facilities that were also part of hotspot P1 of the primary analysis, suggesting these facilities contributed most to the high rate of recent infection in their respective localities. Hotspots S2 and S3 included facilities with significantly elevated rates that were not identified in the primary analysis. Median age (range = 25–30 years) and percentage of females (range = 60%–78%) among hotspots in the secondary analysis were similar to hotspots in the primary analysis.

**TABLE 2:** Characteristics of persons with recent HIV infection in geospatial transmission hotspots among health facilities that implemented surveillance for recent HIV infection — Malawi, October 2019−March 2020

District*	Transmission hotspot rank^†^	No. of facilities (radius)	No. of persons at risk for HIV	Recent infection rate (per 100,000 population)^ §^	No. of observed recent infections^¶^	No. of expected recent infections**	RR^††^ (p-value)	Median age, yrs (range)^ ¶^	% Aged <30 yrs^¶^	% of females^¶^
**Primary analysis**										
Blantyre	1	4 (10.2 km)	4,699	575	27	9	3.1 (<0.001)	26 (15–38)	55.6	81.5
Machinga and Zomba	2	4 (16.1 km)	10,365	376	39	21	2.0 (0.018)	26 (18–45)	61.5	79 5
Blantyre	3	1 (—)	1,223	818	10	2	4.2 (0.025)	30 (19–44)	50.0	60.0
**Secondary analysis**										
Blantyre	1	2 (2.1 km)	2,959	608	18	6	3.1 (<0.001)	29 (18–36)	50.0	77.8
	2	1 (—)	1,223	818	10	3	3.9 (0.005)	30 (19–44)	50.0	60.0
	3	1 (—)	3,406	470	16	7	2.3 (0.048)	25 (19–60)	62.5	68.8

**Abbreviation:** RR = relative risk.

* Spatial analyses identified six transmission hotspots: three in the primary analysis and three in the secondary analysis.

^†^ Ranked by probability of occurrence based on log-likelihood.

^§^ Cases per 100,000 population. Denominator for rate calculation was total persons at risk for HIV, calculated as the sum of recent infections observed and total negative HIV test results.

^¶^ Among recent infections included in a cluster.

** Expected number of recent infections in facilities included in a cluster based on the rate of infection across all facilities.

^††^ RR for recent infection among persons tested within a cluster.

**FIGURE:**
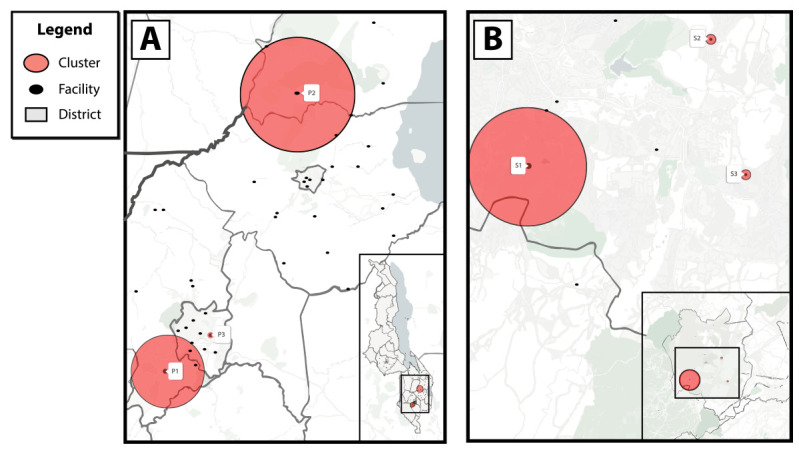
Geospatial transmission hotspots of recent HIV infection among health facilities implementing recent HIV infection surveillance in (A) five districts in Malawi and (B) Blantyre district, Malawi* — October 2019−March 2020 * The primary analysis (A) in five districts (Blantyre, Lilongwe, Machinga, Mangochi, and Zomba) in Malawi with a 20-km (12.4-mi) maximum cluster radius identified three HIV transmission hotspots (P1 = Blantyre, P2 = Machinga and Zomba, P3 = Blantyre [one facility]); a secondary analysis (B) focused on Blantyre district alone with a 5-km (3.1-mi) maximum cluster radius identified three additional HIV transmission hotspots (S1, S2, S3 = all Blantyre district).

## Discussion

This report describes demographic characteristics of persons with recent HIV infection and identifies geospatial hotspots of health facilities with significantly higher rates of recent HIV infection across five districts in Malawi. These findings were consistent with the last national HIV household survey that estimated high HIV incidence in persons aged 15–24 years and females (all ages) (*7*). Location of the most likely hotspots in Blantyre district, a primarily urban district, was consistent with previous evidence from household surveys conducted in 2016 identifying Blantyre as an area with high HIV prevalence and lower rates of viral suppression (*7*). Previous evidence indicated that primarily rural Machinga and Zomba districts were also areas with high prevalence but higher viral load suppression; hotspots of recent infections suggested there might still be significant transmission (*8*). Investigations of these hotspots should seek to understand variation in transmission dynamics between urban and rural areas that may warrant implementation of a tailored response and program strengthening efforts. High proportions of females and similar median ages also warrant further investigation into factors contributing to recent transmission in these subpopulations and potential delayed diagnoses or gaps in HIV services for males (*7*).

Findings from this analysis underscore important considerations when examining recent HIV infection surveillance data. Although Blantyre district had a lower percentage of recent infections among new HIV diagnoses (2.4%) than did the overall analysis population percentage (3.3%), geospatial analysis identified hotspots with significantly higher rates of recent infection that might have otherwise gone unrecognized. This supports the importance of using the number of recent infections in the numerator and total recent infections plus total negative HIV tests (total at risk) in the denominator to identify hotspots of increased transmission (*9*). In addition, triangulation of various surveillance data sources and indicators of recent infection is important to understand the true prevalence and transmission of HIV across time and space. Moreover, geographic analyses can be conducted at various levels rather than just administrative borders (e.g., district).

The findings in this report are subject to at least five limitations. First, analysis was limited to five districts in Malawi, thus results might not be generalizable to other settings. Second, although only persons reporting a new HIV diagnosis were eligible for recent infection surveillance, in the absence of unique identifiers and case surveillance, it might not have been possible to determine whether a person had previously received an HIV diagnosis. Third, focusing only on HIV diagnoses overlooks persons with HIV who do not know their status or have not enrolled in treatment. Fourth, HIV testing frequency and behavior might vary across populations. For this analysis, mapping was done using health facility location, which might not reflect client residence, where transmission occurred, or population mobility. Future analyses could map residential-level hotpots, while protecting client privacy. Finally, performance of the test used to identify recent infections was assumed to be similar across all facilities.

Surveillance for recent HIV infection can help identify trends across sub-populations, map geographic areas where transmission has occurred in the past year, detect hotspots including facilities with higher-than-expected rates of recent infection, and guide prevention activities (*10*). After identifying a potential hotspot, investigations can include triangulation of surveillance and HIV program data to examine data quality, collection and reporting issues, programmatic gaps, and factors that elevate risk for infection. Hotspot investigation information could be used to tailor HIV testing, prevention and treatment to ultimately interrupt transmission.

**Summary**

**What is already known about this topic?**

A novel HIV infection surveillance initiative was implemented in Malawi to collect data on recent HIV infections among new diagnoses to characterize the epidemic and guide the public health response.

**What is added by this report?**

Higher proportions of recent infections were identified among females, persons aged <30 years, and clients at maternal and child health and youth clinics. Spatial analysis identified three hotspots of health facilities with significantly higher rates of recent infection than expected across five districts.

**What are the implications for public health practice?**

Geospatial analysis of recent HIV infection surveillance data can identify potential transmission hotspots. This information could be used to tailor program activities to strengthen HIV testing, prevention, and treatment services and ultimately interrupt transmission.

## Footnotes

* As defined by the HIV-1 recent infection surveillance using point-of-care test for recent infection in Malawi protocol.

†
https://www.unaids.org/en/regionscountries/countries/malawi

§
https://trace-recency.org/ufaqs/what-is-a-rapid-test-for-hiv-1-recent-infection-rtri/

¶ Specified as no geographic overlap criteria for reporting hierarchical clusters.

** 45 C.F.R. part 46; 21 C.F.R. part 56.

†† Rural and urban classification based on European Commission’s Global Human Settlement categorization.
